# Linezolid-resistant enterococci in Polish hospitals: species, clonality and determinants of linezolid resistance

**DOI:** 10.1007/s10096-017-2934-7

**Published:** 2017-02-14

**Authors:** I. Gawryszewska, D. Żabicka, W. Hryniewicz, E. Sadowy

**Affiliations:** 1 Department of Molecular Microbiology, National Medicines Institute, Chełmska 30/34, 00-725 Warsaw, Poland; 2 Department of Epidemiology and Clinical Microbiology, National Medicines Institute, Chełmska 30/34, 00-725 Warsaw, Poland

## Abstract

The significant increase of the linezolid-resistant enterococci (LRE) has been observed in Polish hospitals since 2012 and our study aimed at elucidating the possible reasons for this phenomenon. Polish LRE isolates were analysed by multilocus-sequence typing (MLST) and multiple locus variable-number tandem repeat (VNTR) analysis (MLVA), polymerase chain reaction (PCR) and PCR-restriction fragment length polymorphism (PCR-RFLP) to establish clonal relatedness and mechanism of linezolid resistance, respectively. Fifty analysed LRE (2008–2015) included mostly *Enterococcus faecium* (82%) and *Enterococcus faecalis* (16%). *Enterococcus faecium* belonged to the hospital-adapted lineages 17/18 and 78, while *E. faecalis* isolates represented ST6, a hospital-associated type, and ST116, found in both humans and food-production animals. The G2576T 23S rRNA mutation was the most frequent (94%) mechanism of linezolid/tedizolid resistance of LRE. None of the isolates carried the plasmid-associated gene of Cfr methyltransferase, whereas *optrA*, encoding the ABC-type drug transporter, was identified in two *E. faecalis* isolates. In these isolates, *optrA* was located on a plasmid, transferable to both *E. faecium* and *E. faecalis*, whose partial (36.3 kb) sequence was 100% identical to the pE394 plasmid, identified previously in China in both clinical and farm animal isolates. The *optrA*–*E. faecium* transconjugant displayed a significant growth deficiency, in contrast to the *optrA*–*E. faecalis*. Our study indicates the role of mutation acquisition by hospital-adapted clones of enterococci as a major driver of increasing resistance to linezolid and tedizolid. Transferability and apparent lack of a biological cost of resistance suggest that *E. faecalis* may be a natural reservoir of *optrA*, an emerging mechanism of oxazolidinone resistance.

**Electronic supplementary material:**

The online version of this article (doi:10.1007/s10096-017-2934-7) contains supplementary material, which is available to authorized users.

## Introduction

Although enterococci represent generally harmless commensals of humans and animals, they have recently become a common cause of hospital-associated infections (HAIs). The majority of enterococcal HAIs are caused by high-risk enterococcal clonal complexes (HiRECCs) of the two clinically relevant species, *Enterococcus faecalis* and *Enterococcus faecium*. HiRECCs of *E. faecalis* include clonal complexes (CCs) 6 (also known as CC2), 9 and 87 significantly over-represented among hospital strains [1, 2]. Almost all clinical isolates of *E. faecium* were initially included in CC17, subsequently divided into lineages 17, 18 and 78 [3]. The Bayesian analysis of population structure (BAPS) clustered nosocomial isolates into two groups, BAPS 2-1 and BAPS 3-3, corresponding to lineages 78 and 17/18, respectively [4].

The increasing prevalence of enterococci, resistant to important anti-enterococcal drugs, such as aminoglycosides and glycopeptides, currently represents a great challenge for clinicians. Linezolid was the first antimicrobial agent of the class of oxazolidinones introduced to clinical use to treat infections caused by multidrug-resistant aerobic Gram-positive bacteria, including vancomycin-resistant enterococci (VRE) [5]. Linezolid inhibits protein synthesis by binding the central loop of domain V in the 23S rRNA in bacterial ribosome [6] and in enterococci, the G2576T (*Escherichia coli* numbering) mutation in the 23S rRNA gene(s) was, thus far, the predominant mechanisms responsible for the loss of susceptibility to this compound. Other mechanisms of linezolid resistance include methylation of the A2503 residue in 23S rRNA by the Cfr methyltransferase and mutations in the L3 and L4 ribosomal proteins (reviewed recently in [7]). Recently, an efflux mechanism of resistance to oxazolidinones and phenicols was found in both *E. faecalis* and *E. faecium* [8], determined by the plasmid-borne *optrA* gene, responsible for the production of the ABC-type transporter OptrA.

Linezolid-resistant enterococci (LRE) as hospital alert pathogens are collected by the National Reference Centre for Susceptibility Testing (NRCST), located at the National Medicines Institute in Warsaw, Poland. Since 2012, LRE have been increasingly reported to the NRCST. Thus, we performed a study with the aim to characterise recent Polish clinical isolates of LRE with respect to their species composition, susceptibility phenotypes to other antimicrobials, mechanisms of linezolid resistance and clonal relatedness. The prevalence of *optrA*, its genetic environment, transferability and associated fitness cost were of our special interest due to their relevance for the spread of linezolid resistance in enterococci. To our knowledge, this is the first report of such data for LRE from Central-Eastern Europe and one of a very few showing the importance of the transferable *optrA* determinant.

## Materials and methods

### Species identification, antimicrobial susceptibility testing and analysis of vancomycin resistance determinants

Fifty clinical LRE isolates (one isolate per patient) were collected by the NRCST during the period from September 2008 till the end of December 2015 in passive surveillance. The isolates were obtained from 20 hospital settings, located in 12 Polish cities (Supplementary Fig. A1). Species identification was performed using standard microbiological methods and, for *Enterococcus avium*, by multilocus sequence analysis (MLSA) [9]. Total DNA was extracted using Genomic DNA Prep Plus (A&A Biotechnology, Gdynia, Poland). Isolates were stored at −80 °C until further analysis. Antimicrobial susceptibility of the isolates to daptomycin and tedizolid was evaluated with the use of stripes with predefined antibiotic concentrations (bioMérieux, Marcy-l’Etoile, France and Liofilchem, Roseto degli Abruzzi, Italy, respectively) and for the remaining antimicrobials (Table 1) by the broth microdilution method, according to the Clinical and Laboratory Standards Institute (CLSI) guidelines, using reference strains *E. faecalis* ATCC 29212 and *Staphylococcus aureus* ATCC 29213 as quality controls. Minimum inhibitory concentration (MIC) interpretation was conducted according to the European Committee on Antimicrobial Susceptibility Testing (EUCAST) or CLSI clinical breakpoints (when EUCAST breakpoints were not available) and the clinical breakpoint for tedizolid for *E. faecalis* was applied for all enterococcal isolates. The detection of *vanA* and *vanB* was performed by polymerase chain reaction (PCR) [10, 11], with *E. faecium* BM4147 and *E. faecalis* V583 as positive controls, respectively. The Tn*1546* structure was investigated by PCR mapping [12–17].

**Table 1:** Antimicrobial susceptibility of Polish linezolid-resistant *Enterococcus faecium* and *Enterococcus faecalis*

Antimicrobial agent/phenotype	*E. faecium*			*E. faecalis*		
	R (%)	MIC_50_	MIC_90_	R (%)	MIC_50_	MIC_90_
Linezolid	41 (100)	16	64	8 (100)	16	32
Tedizolid	41 (100)	4	>32	8 (100)	4	16
Penicillin	41 (100)	256	>256	1 (12.5)	4	16
Ampicillin	41 (100)	256	>256	0	0.5	1.0
HLGR	33 (80.5)	>1024	>1024	7 (87.5)	>1024	>1024
HLSR	31 (75.6)	2048	>2048	8 (100)	>2048	>2048
Vancomycin	37 (90.2)	>256	>256	0	2.0	4.0
Teicoplanin	34 (82.9)	64	256	0	0.25	0.5
Tetracycline	12 (29.3)	0.25	32	8 (100)	64	128
Erythromycin	31 (75.6)	256	>256	8 (100)	>256	>256
Tigecycline	0	0.25	0.25	0	0.25	0.25
Ciprofloxacin	41 (100)	256	>256	8 (100)	128	128
Chloramphenicol	22 (53.6)	32	64	6 (75.0)	64	128
Rifampin	39 (95.2)	128	>256	6 (75.0)	8	64
Daptomycin	0	2	4	0	1	2
Quinupristin–dalfopristin	0	0.5	1	nd	nd	nd

*R* resistant; *HLGR* high-level gentamicin resistance; *HLSR* high-level streptomycin resistance; *nd* not determined; a single isolate of *E. avium* was resistant to penicillin, ampicillin, streptomycin, tetracycline and erythromycin, and had MIC values for linezolid = 16 ml/L and tedizolid = 4 mg/L

### Molecular typing of isolates

The clonal relatedness of *E. faecium* and *E. faecalis* was investigated using multilocus sequence typing (MLST) [1, 3] and the MLST database (http://pubmlst.org/; 1st February 2016, date last accessed). Sequence types (STs) were assigned to CCs with comparative eBURST analysis against the whole databases of *E. faecium* and *E. faecalis* (http://eburst.mlst.net/; 1st February 2016, date last accessed). *E. faecium* isolates were additionally investigated using multiple locus variable-number tandem repeat (VNTR) analysis (MLVA; http://www.umcutrecht.nl/en/Research/Miscellaneous/MLVA-typing; 1st August 2016, date last accessed).

### Detection and analysis of linezolid resistance determinants

Isolates were examined for the presence of the G2576T mutation in the 23S rRNA genes by sequencing [18] and PCR-restriction fragment length polymorphism (PCR-RFLP) analysis with *Nhe*I [19] of individual copies of 23S rRNA genes of *E. faecium* and *E. faecalis* (primer sequences available upon request). The *rplC* and *rplD* genes encoding ribosomal proteins L3 and L4, respectively, were sequenced [20] and compared to the wild-type sequences from *E. faecalis* ATCC 29212 (CP008816.1), *E. faecium* DO (CP003583.1) and *E. avium* ATCC 14025 (ASWL01000001.1) strains. The *cfr* gene was searched for by PCR [21] using DNA of a *cfr*-positive clinical isolate of *S. aureus* from the laboratory collection as a control (unpublished data) and *optrA* and *repB*_pE349_ were detected by PCR with primers designed in the study (5′-TCAACCTTGAAAGGGGACAG/AGCCAAGAGCAGTTCTGACC-3′ and 5′-TGAATTAGCAGTCGCCAGTTTAG/TTACCGTTGGCAAATATTGTGTG-3′, respectively). The localisation of *optrA* was established with pulsed-field gel electrophoresis (PFGE)-S1/hybridisation analysis. To this end, total genomic DNA was purified in agarose plugs [10], subjected to S1 nuclease digestion (Takara Bio, Japan) and separated by PFGE [22], followed by blotting onto the Hybond-N+ membrane (GE, Healthcare, Buckinghamshire, UK) and hybridisation with the *optrA* probe, using the Amersham ECL Random-Prime Labelling and Detection System (GE Healthcare).

### Conjugation experiments

Transferability of linezolid resistance was studied for the *optrA*-positive isolates using a highly efficient conjugation protocol [23] with *E. faecalis* OG1RF and *E. faecium* 64/3 as recipients.

### Genomic sequencing and plasmidome of *optrA*–*E. faecalis*

Whole-genome sequencing (WGS) was performed with Ion Torrent PGM (Thermo Fisher Scientific, Waltham, MA, USA) or MiSeq (Illumina, San Diego, CA, USA) as an external service. Obtained reads were assembled using the CLC Genomic Workbench v.8 (CLC bio, Aarhus, Denmark) and joining of contigs was performed with PCR (primer sequences available upon request), followed by sequencing of the products. The presence of *rep* genes, unique and characteristic for families 1–19, was either analysed by PCR [24] or deduced from WGS data using the BLAST function in the CLC software.

### Growth experiments

The growth ratios of *optrA* transconjugants and recipient strains as a measure of the bacterial fitness and plasmid cost [25] were determined by culturing tested strains in BHI broth with and without linezolid (8 mg/L) overnight, respectively, diluting to OD 0.01 in BHI broth without antibiotics and growing at 37 °C with shaking for 10 h. OD_600_ measurements were taken every 30 min. The test was done twice, in three biologically independent replicates. The number of colony-forming units (CFUs) in cultures of recipients and transconjugants from the beginning and end of the test (t_0_ and t_10_, respectively) was established by plating appropriate dilutions on BHI agar and the enumeration of colonies.

### Nucleotide accession numbers

The nucleotide sequence of *rplD* of *E. avium*, the complete sequence of *optrA*-plasmid 1 from the KIEL *E. faecalis* isolate and a partial sequence of plasmid 2 from the same isolate were deposited in GenBank (accession numbers KX255697, KY513280 and KY513281, respectively).

## Results

### Bacterial isolates: epidemiological data, species, antimicrobial susceptibility and clonal relatedness

LRE were reported as single cases for 13 hospitals, while seven hospitals, located in Warsaw, Wroclaw, Poznan and Gdansk, sent up to 12 LRE each (Table 2, Supplementary Fig. A1). The most prevalent species, *E. faecium* (41 isolates, 82%), occurred in 16 hospitals, while *E. faecalis* (8 isolates, 16%) was obtained in four centres. One isolate was identified as *E. avium*. The first LRE was sent to the NRCST in September 2008, and, since 2012, the number of LRE per year has been generally increasing, in parallel to the number of affected hospitals (Table 2). Nearly half of the isolates were obtained from colonised patients (21; 42%); 15 and 14 isolates were from invasive and non-invasive infections, respectively. Linezolid MIC values ranged from 8 to 128 mg/L and all isolates were resistant to tedizolid (Table 1). All *E. faecium* and *E. faecalis* isolates were resistant to ciprofloxacin, susceptible to tigecycline and daptomycin and, in the case of *E. faecium*, to quinupristin–dalfopristin. Additionally, all *E. faecium* isolates displayed resistance to penicillin and ampicillin. In both species, a high prevalence of high-level aminoglycoside resistance (HLAR) was observed. Vancomycin resistance occurred only among *E. faecium* (37 isolates, 90.2%), mediated by the *vanA* and *vanB* gene clusters (34 and three isolates, respectively). All LRE exhibited the MDR phenotype (resistance to three or more classes of antimicrobials). Six *E. faecalis* isolates belonged to ST6, specific for HiRECC 6, and the remaining two isolates represented ST116 from CC116. *Enterococcus faecium* isolates represented eight related MLVA types (MTs) and 11 STs, characteristic for nosocomial lineages 17/18 and 78 (Table 2). Typing of Tn*1546* of 20 *vanA*-positive *E. faecium* of MT12/ST117 from seven centres identified three transposon variants, A, B and C (Table 2).

**Table 2:** Centre, year of isolation, typing data and resistance determinants of Polish linezolid-resistant enterococci (LRE) hospital isolates

A.*E. faecium*															
Centre	Year of isolation^a^	ST	Lineage	MT	MIC (mg/L)	Linezolid resistance determinants									Tn*1546* type^d^
					Linezolid	Tedizolid	1^b^	2^b^	3^b^	4^b^	5^b^	6^b^	Mutated copies of 23S rDNA	*optrA*	
GDA	2013 (1)	17	17/18	7	128	>32	−	−	−	+	−	+	2	−	
	2013 (1)	117	17/18	527	128	>32	+	−	−	−	+	+	3	−	
	2013 (2)	202	17/18	1	16–32	8–16	+	−	−	−	+	+	3	−	
	2013 (7)	117	17/18	12	32–64	8–32	+	−	−	−	+	+	3	−	A
	2014 (1)	117	17/18	12	32	4	+	−	−	−	+	+	3	−	A
LEG	2011 (1)	877	17/18	12	16	>32	−	−	−	+	+	+	3	−	
LODZ1	2012 (1)	117	17/18	12	8	4	−	−	−	+	−	+	2	−	A
LODZ2	2015 (1)	117	17/18	12	8	2	−	−	+	+	−	+	3	−	A
POZ1	2011 (1)	202	17/18	11	8	4	−	−	−	−	+	+	2	−	
POZ2	2012 (4)	117	17/18	12	8	8	−	−	+	−	+	+	3	−	
					8	8	−	−	+	−	+	+	3	[+]	
					8	1	−	+	+	−	+	+	4	−	
					8	4	−	−	+	−	+	+	3	[+]	
POZ3	2015 (1)	18	17/18	10	32	4	+	−	−	+	+	+	4	−	
SOC	2014 (1)	117	17/18	12	32	1	−	−	−	+	−	+	2	−	B
WLO	2015 (5)	203	78	159	16–32	2–4	−	−	+	−	+	+	3	−	
WRO1	2013 (1)	17	17/18	1	16	4	−	−	−	+	−	+	2^c^	[+]	
WRO2	2014 (2)	117	17/18	12	32	2	−	−	+	−	−	+	2	−	C
		117	17/18	12	16	16	+	+	+	+	−	+	5	−	C
WAR1	2013 (1)	78	78	282	16	16	−	+	−	−	+	+	3	−	
WAR2	2013 (1)	412	78	159	8	2	−	−	−	+	−	−	1	−	
WAR3	2011 (1)	880	78	1	16	8	−	−	+	−	+	+	3	−	
	2011 (1)	80	17/18	1	8	4	−	−	−	−	+	+	2	−	
	2014 (1)	80	17/18	1	32	4	−	−	−	−	−	−	0	−	
	2014 (1)	117	17/18	159	8	2	−	−	−	−	−	+	1^c^	−	
WAR4	2010 (1)	78	78	159	16	4	−	+	−	+	+	+	4	−	
WAR5	2008 (1)	192	78	159	16	2	−	−	−	−	+	+	2	−	
	2014 (1)	117	17/18	12	32	16	+	−	+	+	+	+	5	−	C
	2015 (2)	117	17/18	12	128	>32	+	−	+	+	+	+	5	−	A
B. *E. faecalis*															
Centre	Year of isolation ^a^	ST	CC	MIC (mg/L)	Linezolid resistance determinants										
				Linezolid	Tedizolid	1^b^	2^b^	3^b^	4^b^	Mutated copies of 23S rDNA	*optrA*				
POZ1	2012 (4)	6	6	8–16	4–8	+	+	−	−	2	−				
	2012 (1)	6	6	32	16	+	+	−	+	3	−				
RZE	2015 (1)	6	6	32	4	+	−	−	+	2	−				
KIEL	2012 (1)	116	116	32	16	−	−	−	−	0	+				
BYD	2013 (1)	116	116	16	4	−	−	−	−	0	+				

*ST* sequence type; *MT* MLVA type; *CC* clonal complex; *[+]* indicates three *E. faecium* isolates that lost *optrA* determinant upon storage; centres: *GDA* Gdansk; *LEG* Legnica; *LODZ* Lodz; *POZ* Poznan; *SOC* Sochaczew; *WLO* Wloclawek; *WRO* Wroclaw; *WAR* Warsaw; *KIEL* Kielce; *BYD* Bydgoszcz; *RZE* Rzeszow

^a^Number of isolates in parentheses

^b^The presence of G2576T mutation revealed by PCR-RFLP analysis of individual copies of 23S rDNA

^c^Mutations detected only when particular copies of the 23S rRNA gene were analysed separately

^d^The A- and B-types of Tn*1549* carried IS*1251* between *vanS* and *vanH*, and lacked ORF1 and both ORF1 and ORF2, respectively; the C-type had no IS and was truncated at the 5′ end up to *vanS*

### Detection and analysis of linezolid resistance determinants

**Fig. 1:**
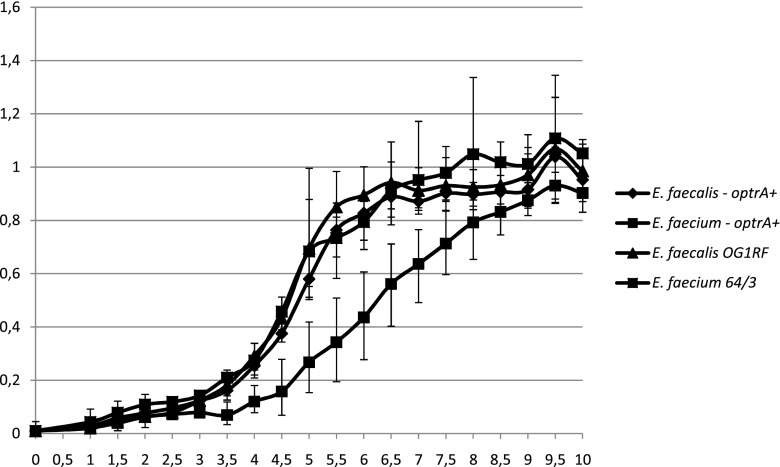
Growth curves of *Enterococcus faecalis* OG1RF and *Enterococcus faecium* 64/3 and their *optrA* transconjugants

Sequencing of the 23S rRNA genes and the PCR-RFLP analysis of individual copies of 23S rDNA revealed the presence of the G2576T mutation for 47 isolates (94%); the remaining single isolate of *E. faecium* and two isolates of *E. faecalis* harboured the wild-type 23S rRNA genes (Table 2). The number of mutated copies of the 23S rRNA gene ranged from one to five out of six in *E. faecium* and from two to three out of four in *E. faecalis*. All isolates harboured the wild-type *rplC* gene. Mutation in the *rplD* gene was observed only for *E. avium*, where the T511G substitution resulted in the S171A change in the L4 deduced amino acid sequence. None of the isolates carried the *cfr* gene. Initially, *optrA* was detected in five isolates (10%), including two *E. faecalis* and three *E. faecium*. In the case of the three latter isolates, the gene was lost upon storage; thus, detailed analyses of *optrA* localisation and transferability could be continued only for *E. faecalis*. A remaining single isolate of *E. faecium* (linezolid MIC = 32 mg/L) did not contain any of the resistance determinants studied. The PFGE-S1 analysis of *optrA*-positive *E. faecalis* isolates showed the presence of two or three plasmids, ranging from <50 to ∼100 kb in size, and the subsequent Southern blot analysis showed the localisation of *optrA* on a plasmid below 50 kb in both cases. *optrA* was transferable by conjugation to both *E. faecalis* and *E. faecium* recipients. *Enterococcus faecium* transconjugants harboured a single plasmid of the size corresponding to the *optrA* plasmid from clinical isolates, while in *E. faecalis* transconjugants, either the *optrA* plasmid was accompanied by another plasmid, also below 50 kb in size, or a single ∼100-kb plasmid was observed. WGS of an *optrA*–*E. faecalis* transconjugant (donor: isolate from 2013 from a BYD hospital) with a ∼100-kb presumably recombinant plasmid yielded five contigs (sequencing coverage 86–140×), absent from the OG1RF genomic sequences (accession no. NC_017316). Two of these contigs were successfully joined by PCR into a 36,331-bp contig, 100% identical to the corresponding part of the conjugative plasmid pE349 carrying *optrA* (accession no. KP399637) [8] and characterised by the presence of the unique *repB*_pE349_ gene. The remaining three contigs showed a partial homology to the region encompassing 1.2–41.6 kb of the pTEF2 plasmid (accession no. AE016831; data not shown). In the donor isolate, PCR analysis revealed the presence of *rep9* and *rep17* genes. In growth experiments, an *optrA*–*E. faecium* transconjugant was characterised by a significantly delayed entrance into the logarithmic phase of growth, compared to the *E. faecium* 64/3 recipient, as well as to an *optrA*–*E. faecalis* transconjugant and the *E. faecalis* OG1RF recipient (Fig. 1). WGS of the other *optrA* clinical isolate (obtained in 2012 in a KIEL hospital) demonstrated that two contigs (35,000 bp and 1526 bp, coverage 178× and 329×, respectively) had sequences identical to pE349. These two contigs were successfully joined by PCR and sequencing of the products into a single circular plasmid, 36,331 bp in size (Supplementary Fig. 2A). This plasmid was also transferable by conjugation to both *E. faecalis* and *E. faecium*. The BLAST analyses of genomic sequences of the KIEL *E. faecalis* isolate revealed solely the presence of a representative of *rep9* family, with the highest homology (94.0–96.6%) to *prgW* from pBEE99 (GU046453; [26]) and to *repA* genes of pMG2200 (AB374546; [27]) and pTEF2 (AE016831; [28]). This gene was located in a 20,525-bp contig (coverage 105×). This contig, presumably representing a part of a plasmid, showed similarity to pheromone-responsive plasmids, such as pBEE99 and to some Gram(+) integrative-conjugative elements, such as Tn*6248* (KP834592), found in a pig isolate of *E. faecium* in China (Supplementary Fig. 2B).

## Discussion

Linezolid is an important agent in the treatment of serious infections caused by multidrug-resistant Gram-positive organisms, including methicillin-resistant *S. aureus* (MRSA) and VRE. The first case of LRE in Poland occurred in July 2003, shortly after the introduction of linezolid for treatment in our country in February 2002 [29]. Although LRE remain relatively rare in Poland [30], their increasing number is reported to the NRCST, along with a growing number of affected hospitals. While in systematic surveillance a stable low prevalence of linezolid resistance in Gram-positive bacteria, including enterococci, is observed [7], some other studies show a local increase of LRE, e.g. in Germany [31], similar to the situation observed in Poland. In our study, over 70% of LRE were isolated from patients of intensive care units (ICUs) and haematology wards, which are wards with the highest use of antimicrobials, including linezolid. LRE occurred mostly as sporadic cases, in agreement with the previous observations about the nature of LRE emergence [32]. Nevertheless, in some centres, the epidemic spread of LRE was observed, as indicated by a similar time of isolation, the same genotype and mechanism of resistance. Linezolid resistance was identified almost exclusively in two enterococcal species, which are the most frequent causes of enterococcal HAIs, *E. faecalis* and *E. faecium*. The linezolid-resistant *E. faecium* showed much higher prevalence of vancomycin resistance (90.2%), than typically found in HAIs in Polish hospitals for this species (7.1%) [30]. The analysis of clonality, resistance determinants and the Tn*1546* structure indicated possible VRE/LRE outbreaks and even presumable transfer(s) among hospitals. The combination of these two resistance phenotypes in the background of multiresistant epidemic hospital meroclone is especially worrisome due to the lack of effective treatment options, and highlights the importance of active surveillance and infection control procedures.

The G2576T mutation within the domain V of 23S rRNA was the most frequent mechanism of linezolid resistance developed by Polish LRE, as frequently reported for enterococcal clinical isolates [7]. None of the isolates, however, harboured the G2576T mutation in all copies of 23S rDNA, which indicates that lack of the wild-type copy might impose too big a fitness cost for the cell [33]. The second mutational mechanism of resistance, was the T511G mutation in *rplD* (the S171A substitution in the L4 ribosomal protein) identified in our study for *E. avium*. The S/P mutation at the same position in a clinical *E. avium* isolate also from Poland constituted the only mechanism responsible for its elevated MIC of linezolid [7]. The only non-mutational mechanism of linezolid resistance detected in our study was the presence of ABC transporter OptrA [8]. Another linezolid resistance determinant, *cfr*, was not found, although its presence was observed for both *E. faecalis* and *E. faecium* [20, 34]. The *optrA* gene was first identified on the pE349 plasmid [8] and detected in human and animal *E. faecalis* and *E. faecium* from China, Malaysia, Austria, Ireland and Italy [8, 35–37]. In our study, the *optrA* gene was the only determinant of linezolid resistance detected in two epidemiologically unrelated non-HiRECC *E. faecalis* of ST116. We also observed a loss of this determinant from three isolates of *E. faecium*, which additionally carried the G2576T mutation. A loss of conjugative resistance plasmids was reported by others [25]. Such instability might explain the generally much lower prevalence of *optrA* in *E. faecium* compared to *E. faecalis* [8, 38], and would be in agreement with the fitness cost imposed by an *optrA* plasmid on *E. faecium* transconjugant, observed in this study. The *optrA*-carrying plasmid was transferable from *E. faecalis* to both *E. faecium* and *E. faecalis* recipients, alone or with a second plasmid, respectively. The *optrA* plasmids of *E. faecalis* characterised so far are typically conjugative [8]. It is tempting to speculate about a possible source of the *optrA* gene, located on pE349, for clinical enterococci in Poland. As previously suggested, *E. faecalis* ST116, characteristic for isolates from both human infections as well as food-producing animals and retail meat [8, 39], may act as a vehicle of antimicrobial resistance between environment and hospital, similarly to vancomycin-resistant *E. faecalis* from imported turkey meat and clinical isolate, which also shared this ST [39]. Moreover, three isolates of *E. faecalis* from poultry, carrying pE349-like plasmids with the *optrA* gene, were reported recently from Columbia [40], indicating a global spread of *optrA* plasmids and supporting the potential role of an animal reservoir. Thus, a food-borne *E. faecalis* with pE349 could serve as a potential source of *optrA* for nosocomial enterococci, raising a new aspect for food safety.

In conclusion, we performed an extensive analysis of linezolid- and tedizolid-resistant isolates of *Enterococcus* spp., obtained recently from carriage and HAIs in Polish hospitals. The emergence of LRE was predominantly caused by independent de novo resistance development in various enterococcal strains, mostly representing HiRECCs, for which acquisition of linezolid resistance may be a next step in their evolution as hospital ‘superbugs’. While the mutation within the 23S rRNA gene was the most common determinant of resistance, the detection of the multiresistance gene *optrA* among Polish LRE and evidence for its in vitro transferability raises further concerns about the possible decreasing effectiveness of linezolid treatment in the future.

## Electronic supplementary material

Below are the links to the electronic supplementary material.

ESM 1 Distribution of LRE in Poland, 2008–2015. The sizes of the circles are proportional to the number of LRE identified in each city; the number of LRE identified in particular hospitals in a city is indicated within the circles; cities from which *optrA*-positive isolates were submitted are underlined. (PDF 41 kb)

ESM 2 Plasmids from the KIEL *E. faecalis* isolate. A. The structure of the *optrA* plasmid, 100% homologous to pE347 [KP399637]. B. The partial structure of the *rep9* plasmid, with the regions with homology to pBEE99 [GU046453] and Tn*6248* [KP834592] marked by solid and dashed arrows, respectively. Plasmid replication genes in blue, conjugation genes in green, antimicrobial resistance genes in red, other genes/genes of unknown function in yellow. The UGENE software (Okonechnikov K, Golosova O, Fursov M, the UGENE team. Unipro UGENE: a unified bioinformatics toolkit. Bioinformatics 2012 28:1166–1167. doi:10.1093/bioinformatics/bts091) was used to visualise ORFs, followed by manual editing of the figure. (PDF 176 kb)
