# Supplementary figures and images for: Linezolid-resistant enterococci in Polish hospitals: species, clonality and determinants of linezolid resistance
Source: Eur J Clin Microbiol Infect Dis. 2017 Feb 14;36(7):1279–86. doi: 10.1007/s10096-017-2934-7 (PMC5495842; doi:10.1007/s10096-017-2934-7)

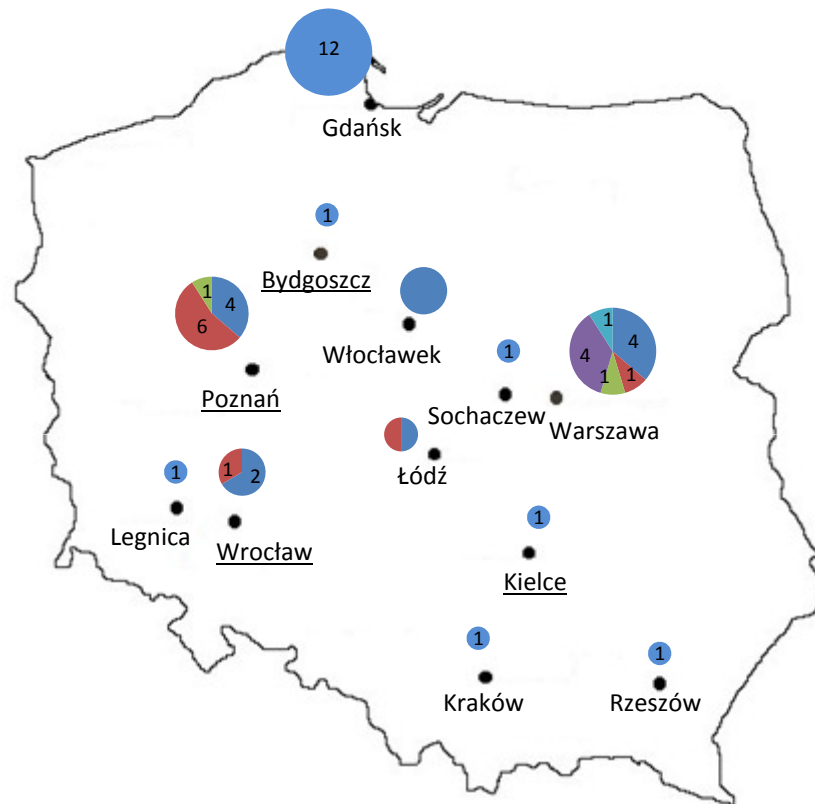

Supplement: Supplementary file 1 — Distribution of LRE in Poland, 2008–2015. The sizes of the circles are proportional to the number of LRE identified in each city; the number of LRE identified in particular hospitals in a city is indicated within the circles; cities from which optrA-positive isolates were submitted are underlined. (PDF 41 kb) [file 10096_2017_2934_MOESM1_ESM.pdf]

A.

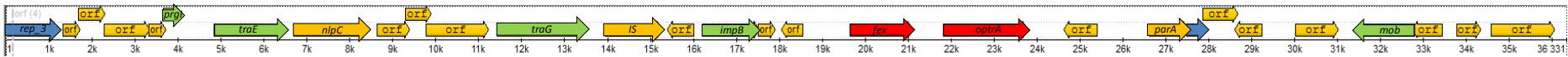

B.

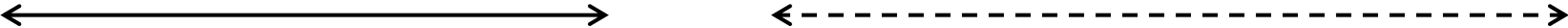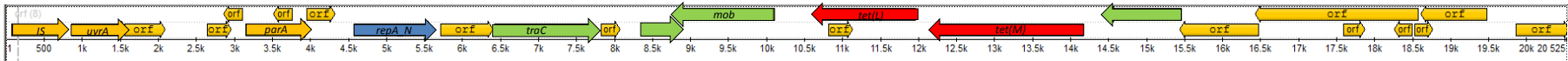

Supplement: Supplementary file 2 — Plasmids from the KIEL E. faecalis isolate. A. The structure of the optrA plasmid, 100% homologous to pE347 [KP399637]. B. The partial structure of the rep9 plasmid, with the regions with homology to pBEE99 [GU046453] and Tn6248 [KP834592] marked by solid and dashed arrows, respectively. Plasmid replication genes in blue, conjugation genes in green, antimicrobial resistance genes in red, other genes/genes of unknown function in yellow. The UGENE software (Okonechnikov K, Golosova O, Fursov M, the UGENE team. Unipro UGENE: a unified bioinformatics toolkit. Bioinformatics 2012 28:1166–1167. doi:10.1093/bioinformatics/bts091) was used to visualise ORFs, followed by manual editing of the figure. (PDF 176 kb) [file 10096_2017_2934_MOESM2_ESM.pdf]
